# Comparison of intact protein and digested peptide techniques for high throughput proteotyping of ApoE

**DOI:** 10.1186/s12014-022-09379-5

**Published:** 2022-11-15

**Authors:** Anthony Maus, Dan Figdore, Dragana Milosevic, Alicia Algeciras-Schimnich, Joshua Bornhorst

**Affiliations:** Department of Laboratory Medicine and Pathology, Division of Clinical Biochemistry and Immunology, Mayo Clinic, 200 First Street SW, Rochester, MN 55905 USA

**Keywords:** Apolipoprotein E, Alzheimer’s disease, Tandem mass spectrometry, High-resolution mass spectrometry

## Abstract

**Introduction:**

Apolipoprotein E (ApoE) genotyping has been shown to have diagnostic value in the evaluation of cardiovascular diseases and neurodegenerative disorders such as Alzheimer’s disease. Although genetic testing is well established for this application, liquid chromatography-mass spectrometry (LC–MS) has the potential to provide a high throughput, low-cost alternative for ApoE evaluation.

**Methods:**

Serum samples were analyzed by peptide, intact protein, and genomic techniques. For peptide analysis, samples were digested with trypsin followed by liquid chromatography-tandem mass spectrometry analysis (LC–MS/MS) using a high-throughput multichannel LC system coupled to a Sciex 7500 mass spectrometer. For intact protein analysis, ApoE was immuno-purified using a monoclonal antibody immobilized on magnetic beads followed by high-resolution LC–MS analysis using an Exploris 480. DNA was extracted and evaluated using Sanger sequencing as a reference method.

**Results and discussion:**

The peptide measurement method produced one discrepant result when compared to genomic sequencing (out of 38 sequenced samples), whereas the intact protein analysis followed by deconvolution resulted in two discrepant results and when the intact protein data was processed with chromatographic integration there were three discrepant results. Therefore, the intact protein method proved slightly less accurate, required longer analysis time, and is substantially more costly, while providing only a 30 min improvement in sample preparation time.

**Conclusions:**

With current MS technology clinical laboratories appear to be better served to utilize trypsin digest sample preparation and LC–MS/MS as opposed to high-resolution LC–MS intact protein analysis techniques for evaluation of ApoE proteotype. Peptide analysis methods are capable of producing accurate results with high throughput and minimal cost.

**Supplementary Information:**

The online version contains supplementary material available at 10.1186/s12014-022-09379-5.

## Introduction

Apolipoprotein E (ApoE) is a 34 kDa protein that is a major component in cholesterol-rich very low density lipoprotein (VLDL) and is of relatively high abundance in some subclasses of high density lipoproteins (HDL), such as HDL_1_ and HDL_c_ [1]. There are three common isoforms of ApoE, known as ApoE2, ApoE3, and ApoE4, corresponding to the respective alleles in the ApoE gene, which have frequencies in the global population of approximately 8%, 78% and 14%, respectively.[2, 3]. These genetic differences result in different amino acids at two positions in the sequence, with ApoE2 possessing cysteine in 112 and 158, ApoE3 has cysteine in position 112 and arginine in 158, and ApoE4 has arginine in both the 112 and 158 positions. The combinations of these alleles give rise to 6 possible genotypes, with both homozygous (E2/E2, E3/E3, E4/E4) and heterozygous (E2/E3, E3/E4, E2/E4) being prevalent at various frequencies in the global population [4].

The ApoE genotype has implications for cardiovascular diseases and neurodegenerative disorders [1, 5]. ApoE2 homozygosity has been shown to be related to type III hyperlipoproteinemia, which ultimately causes premature atherosclerosis [6, 7]. The ApoE4 genotype is associated with increased abundance of low-density lipoproteins and risk of atherosclerosis [7, 8]. The relationship between ApoE genotype and neurodegenerative disorders has also been extensively explored. The presence of ApoE4 protein has been associated with negative ramifications in regards to traumatic brain injury, stroke, frontotemporal dementia, Down syndrome, Parkinson’s disease, and Lewy body disease [1, 9], in addition to having been shown to increase the risk of Alzhiemer’s disease (AD) three–fourfold in heterozygotes and about 9–15 fold in ApoE4 homozygotes compared to non-carriers of the ApoE4 protein [2, 10, 11]. In contrast, several studies show that the ApoE2 genotype reduces the risk of cognitive impairment and AD [10, 12]. One notable example from Reiman et al. indicated that ApoE2 homozygotes have a 66% decrease in AD risk compared to ApoE2/ApoE3 heterozygotes, an 87% reduction relative to ApoE3 homozygotes, and a 99.6% reduction compared to ApoE4 homozygotes [10, 13].

Although the relationship between ApoE genotype, cardiovascular diseases, and neurodegenerative disorders is well established, the diagnostic role of quantitative plasma ApoE concentration measurements are more controversial [5]. Increased ApoE concentrations have generally been shown to be related to increased levels of triglycerides and cholesterol, which are well known drivers of cardiovascular diseases [14–16]. However, some studies indicate a relationship between ApoE concentrations and neurocognitive decline, whereas others do not suggest such a relationship [17–21]. Therefore, at this time no clear clinical benefit for quantitative ApoE testing has been established.

Genetic testing has been utilized as the standard for ApoE classification determination for decades [22, 23]; however, liquid chromatography-mass spectrometry (LC–MS) has the potential to provide a low cost, high throughput alternative to genetic testing with the additional potential benefit of being capable of producing precise and accurate quantitative results in cases where both protein identity and concentration are desired. Several investigations predicated on measurement of trypsin digested peptides have been published [3, 4, 24, 25]. These methods rely on the detection of four target peptides derived from the ApoE protein to determine the proteotype. A particularly relevant example of this methodology was recently published by Brkovic et al. [26]. This peptide analysis method used online sample purification, a cornerstone of clinical LC-tandem mass spectrometry (LC–MS/MS) analyses, and had an analysis time of only 6.5 min. However, the method did use overnight digestion, which would negatively impact turnaround time in a clinical testing environment. Additionally, this method utilized microflow flow rates, which are not widely employed in routine clinical laboratories.

Given the relatively moderate size of ApoE (34 kDa) analysis of the intact protein is an attractive alternative to trypsin digestion. Hu et al. demonstrated this when they performed immuno-purification of ApoE, followed by analysis using matrix-assisted laser desorption/ionization-time of-flight-mass spectrometry (MALDI-TOF–MS) [27]. This intact protein analysis technique is easy to perform and high-throughput; however, the lack of mass or chromatographic resolution of different ApoE isoforms would make interpretation in a routine clinical laboratory challenging.

In this investigation, we aimed to explore the potential of trypsin digestion followed by analysis of peptides using LC separation and a triple quadrupole mass spectrometer, as well as immuno-purification (IP) followed by analysis of the intact ApoE protein using LC separation and a high-resolution Exploris 480 Orbitrap mass spectrometer.

## Materials and methods

### Chemicals

Water was purified using a Barnstead Nanopure system (ThermoFisher Scientific, Waltham, MA). Phosphate Buffered Saline (PBS), tris base, hydrochloric acid, and trifluoroacetic acid (TFA) were purchased from Fisher Scientific. LC–MS grade acetonitrile (ACN), formic acid, isopropanol (IPA), and bovine serum albumin (BSA), were purchased from MilliporeSigma (Burlington, MA). Isotopically labeled peptides used as internal standards (IS) for the four target peptides were synthesized by the Proteomics Core at Mayo Clinic using standard 9-fluorenylmethoxycarbonyl (FMOC) chemistry on a Liberty Blue (CEM Corp. Matthews, NC) peptide synthesizer with methods suggested by the manufacturer.

### Trypsin Digest and low-resolution LC–MS/MS Analysis

First, 25 μL of serum sample was put in a 96 well plate, followed by 25 μL of internal standard, 50 μL of 1 M tris–HCl (pH 8) buffer, and 400 μL of water. Proteins were reduced by adding 50 μL of 100 mM dithiothreitol (MilliporeSigma) and incubated at 48 °C for 45 min. Next, proteins were alkylated by adding 125 μL of 100 mM iodoacetamide (MilliporeSigma) and incubating in the dark for 30 min. Proteins were digested for 1 h at 37 °C following the addition of 100 μL of 1 mg/mL trypsin (Worthington, Lakewood, NJ). The digestion was stopped by the addition of TFA to a final concentration of 0.2%.

LC separation was performed using a Thermo Transcend TLX-4 TurboFlow system. Digests (10 μL) were injected onto a C_8_ cartridge (Phenomenex, Torrance, CA, Item number: AJO-6073) with an internal diameter of 2 mm and a length of 4 mm using the loading pump to deliver 2% B at 1 mL/min for 1 min. Next, the sample was eluted off the cartridge at 100 μL/min for 1.25 min using 40% B, and this eluate was mixed via a tee with the eluting pump flowing 2% B at 400 μL/min prior to loading onto the analytical column. The analytical column was a Kinetex PS-C_18_ with an inner diameter of 3 mm, a length of 50 mm, and 2.6 μm particles (Phenomenex). Separation was performed at 600 µL/min using a gradient from 5%B to 13% B over 6 min. The column was then washed for 3 min and equilibrated at starting conditions for 1 min. Therefore, the total LC method time is 12.5 min, but when multiplexed across the 4 channels, results are produced effectively every 3.125 min.

Tandem mass spectrometry analysis was performed using a Sciex 7500 mass spectrometer. Source conditions and MS/MS parameters can be seen in Additional file 1: Tables S1 and S2, respectively. After the LC–MS/MS measurement, chromatographic peak areas for two fragments from the IS and analyte signals were integrated in Sciex OS. The retention times corresponding to the IS were integrated regardless of analyte signal intensity. The resulting integrated areas were then exported to Microsoft Excel for further processing. The analyte signals were corrected by dividing by the IS peak areas. This IS corrected result was then used to determine positive/negative for a given peptide, which was ultimately used to determine the proteotype.

### Immuno-purification of ApoE and high-resolution LC–MS analysis

Antibody purification was performed using a rabbit monoclonal antibody purchased from ThermoFisher Scientific (catalog #-701,241) coupled to tosyl activated magnetic beads (ThermoFisher Scientific, catalog#-14,204). For coupling, beads are washed with a 0.1 M boric acid (MilliporeSigma) buffer pH 9.5. Antibody was then incubated overnight with the beads at 37 °C in a solution of 500 μL of 0.1 M boric acid buffer pH 9.5 and 500 μL of 3 M ammonium sulphate (MilliporeSigma) in 0.1 M boric acid buffer pH 9.5. After overnight incubation, remaining active sites are blocked using a solution of 5 mg/mL BSA in PBS and incubating for 1 h at 37 °C. Next, the beads were washed three times with 0.1% Tween-20 (MilliporeSigma, catalog #-P-1379) in PBS. After final washing, beads were resuspended in 0.1% Tween-20 in PBS and refrigerated for future use.

When performing immuno-purification of ApoE, 10 μg of bead coupled antibody was added to 500 μL of patient serum followed by a 1.5 h incubation at room temperature. The beads were then washed twice with PBS, twice with water, and eluted with 50μL of 20% acetonitrile, 0.4% TFA in water. This eluate was transferred to autosampler vials and injected without further manipulation.

LC separation was conducted using a ThermoFisher Scientific Vanquish Duo, which allows for separation across two channels; however, the method could be easily transferred to a TLX-4 if throughput improvement were necessary. Sample (20 μL) was injected onto an Agilent (Santa Clara, CA) Poroshell 300SB C_3_ column with a 2.1 mm ID and 75 mm in length (catalog #660,750–909) with the pump delivering 20% B at 400 μL min. Starting conditions were held and separation was performed using a gradient up to 50% B over 20 min. The column was then washed for 7 min and reequilibrated at starting conditions for 3 min. Therefore, the total LC method time is 30 min, but when multiplexed across the 4 channels, individual results are produced effectively every 7.5 min.

High-resolution mass spectrometry analysis was performed using an Exploris 480 mass spectrometer. The instrument scanned from *m/z* 1000–3000 at a resolution of 240,000. Additional mass spectrometry parameters can be seen in Additional file 1: Table S3. The resulting mass spectra were then analyzed using extracted ion chromatogram (XIC) integration and deconvolution of the spectra. XICs were produced in TraceFinder (ThermoFisher Scientific) by summing 6 isotopes from the 8 most abundant charge states (48 total *m/z*), integrated, and exported to Microsoft Excel. We determined this to be the optimal number of isotopes, and adding additional lower intensity isotope signals either did not benefit or negatively impacted S/N of the chromatograms. Spectral deconvolution was performed using Thermo BioPharma Finder. The ApoE proteoforms were chromatographically separated; therefore, the deconvoluted spectra were produced using the “average over selected retention time” function in the software and retention times were set as mean retention times for a given protein ± 0.3 min. Deconvoluted spectral intensities were also exported to Microsoft Excel for box-plot generation and proteotype determination.

### DNA sequencing

Cell free DNA was extracted from residual serum samples with the Qiagen Circulating Nucleic Acid kit (Qiagen, Valencia, CA) using the manufacturer’s protocol. The extraction took approximately 120 min. The status of the sequence variants in the DNA sequence (NM_000041.4) corresponding to ApoE protein was evaluated by Sanger sequencing. A targeted PCR reaction was performed to amplify region containing both SNP’s (rs429358 and rs7412). Universal primers were then used to sequence these regions using ABI BigDye BigDyeTerminators v1.1 (ThermoFisher Scientific). The PCR and sequencing required 155 min. The sequencing traces were analyzed manually with Mutation Surveyor software (SoftGenetics, LLC, State College, PA) (Table 1).

**Table 1:** Peptide detection criteria for ApoE proteotyping

Peptides Detected	ApoE Proteotype
CLAVYQAGAR, LGADMEDVCGR	E2/E2
CLAVYQAGAR, LAVYQAGAR, LGADMEDVCGR	E2/E3
LAVYQAGAR, LGADMEDVCGR	E3/E3
LAVYQAGAR, LGADMEDVCGR, LGADMEDVR	E3/E4
CLAVYQAGAR, LAVYQAGAR, LGADMEDVCGR, LGADMEDVR	E2/E4
LAVYQAGAR, LGADMEDVR	E4/E4

### Samples and human subjects

Random deidentified clinical residual serum samples (n = 276) were obtained for this study. After initial screening by the peptide analysis method as described, 41 of these samples were analyzed using intact protein analysis mass spectrometry-based techniques and by cell-free DNA sequencing. This selected subset was chosen to disproportionately represent atypical ApoE genotypes (see Table 2). The Mayo Clinic Rochester Institutional Review Board approved this study as exempt.

**Table 2:** Results from the various proteotyping techniques utilized in this work compared to genomic sequencing

Patient #	Intact Protein Deconvolution	Intact Protein Chromatogram	Peptide	Genomic Sequencing
1	E3/E4	E3/E4	E3/E4	E3/E4
2	E3/E4	E3/E4	E3/E4	E3/E4
3	E3/E3	E3/E3	E3/E3	E3/E3
4	E3/E4	E3/E4	E3/E4	E3/E4
5	E3/E3*	E3/E3*	E3/E3*	E3/E4*
6	E3/E4	E3/E4	E3/E4	E3/E4
7	E3/E3	E3/E3	E3/E3	E3/E3
8	E3/E3	E3/E3	E3/E3	E3/E3
9	E3/E3	E3/E3	E3/E3	E3/E3
10	E3/E4	E3/E4	E3/E4	E3/E4
11	E2/E3	E2/E3	E2/E3	E2/E3
12	E3/E3	E3/E3	E3/E3	Unable Seq*
13	E3/E4	E3/E4	E3/E4	Unable Seq*
14	E3/E3	E3/E3	E3/E3	E3/E3
15	E3/E3	E3/E3	E3/E3	E3/E3
16	E2/E4	E2/E4	E2/E4	E2/E4
17	E3/E3	E3/E3	E3/E3	E3/E3
18	E3/E3	E3/E3	E3/E3	E3/E3
19	E3/E3	E3/E3	E3/E3	E3/E3
20	E3/E4	E3/E4	E3/E4	E3/E4
21	E2/E3	E2/E3	E2/E3	E2/E3
22	E3/E3	E3/E3	E3/E3	E3/E3
23	E3/E3	E3/E3	E3/E3	E3/E3
24	E3/E3	E3/E3	E3/E3	E3/E3
25	E3/E4	E3/E4	E3/E4	E3/E4
26	E2/E3	E2/E3	E2/E3	E2/E3
27	E2/E3	E2/E3	E2/E3	E2/E3
28	E2/E3	E2/E3	E2/E3	E2/E3
29	E4/E4	E4/E4	E4/E4	Unable Seq*
30	E2/E3	E2/E3	E2/E3	E2/E3
31	E3/E3	E3/E3	E3/E3	E3/E3
32	E3/E3	E3/E3	E3/E3	E3/E3
33	E3/E3	E3/E3	E3/E3	E3/E3
34	E3/E3	E3/E3	E3/E3	E3/E3
35	E3/E3	E3/E3	E3/E3	E3/E3
36	E2/E4	E2/E4	E2/E4	E2/E4
37	E4/E4	E4/E4	E4/E4	E4/E4
38	E2/E4	E4/E4*	E2/E4	E2/E4
39	E4/E4	E4/E4	E4/E4	E4/E4
40	E3/E3	E3/E3	E3/E3	E3/E3
41	E3/E3*	E3/E3*	E3/E4	E3/E4

Three samples were unable to yield sufficient sequencing data. The results from the mass spectrometry-based techniques disagreed with sequencing results for patient 5. The intact methods failed to detect the E4 protein for patient 41, and the chromatogram data processing method did not detect E2 for patient 38

* indicates the Discrepant results

## Results and discussion

**Fig. 1:**
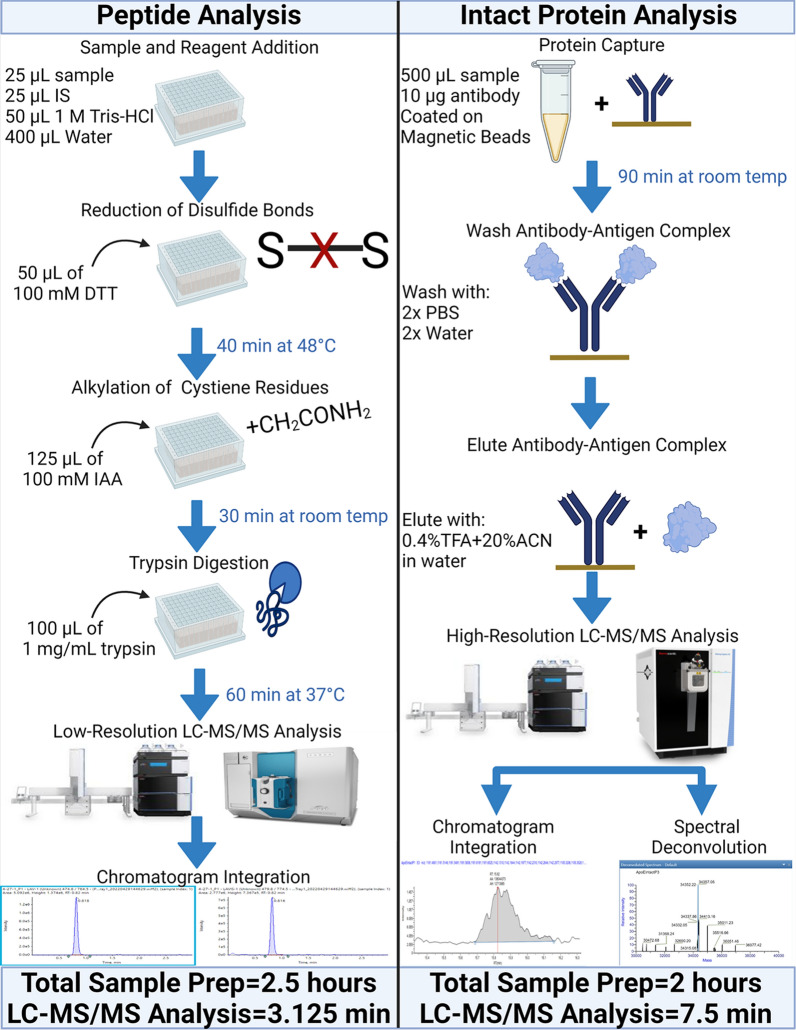
Summary of proteotyping workflows utilized for this study

**Fig. 2:**
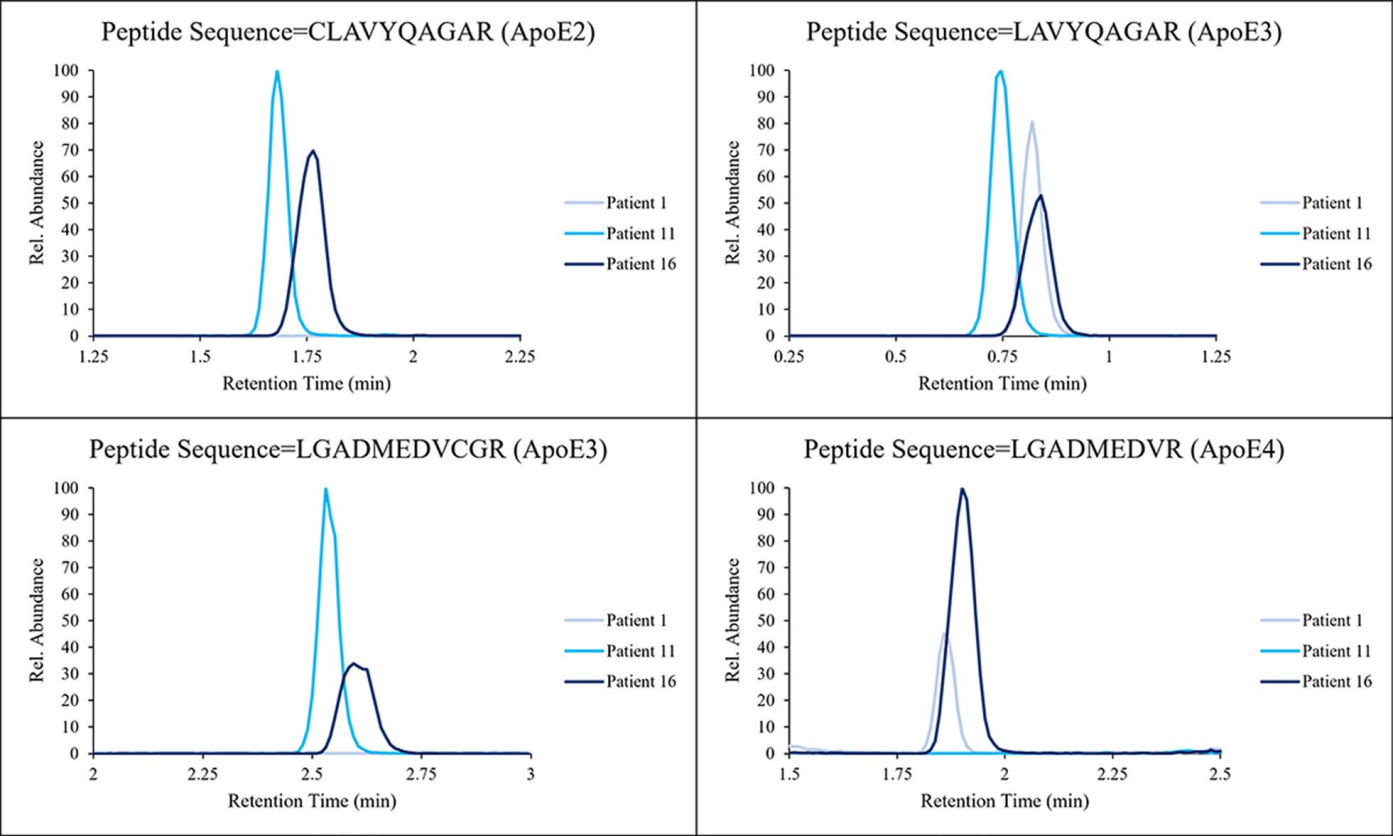
Representative chromatograms of the 4 target peptides from three patients. Patient 1 expressed the ApoE3 and ApoE4 proteins, patient 11 expressed the ApoE2 and ApoE3 proteins, and patient 16 expressed the ApoE2 and ApoE4 proteins. Note that the slight difference in retention time is due to the slight variation across the four lc channels on the multiplex system

**Fig. 3:**
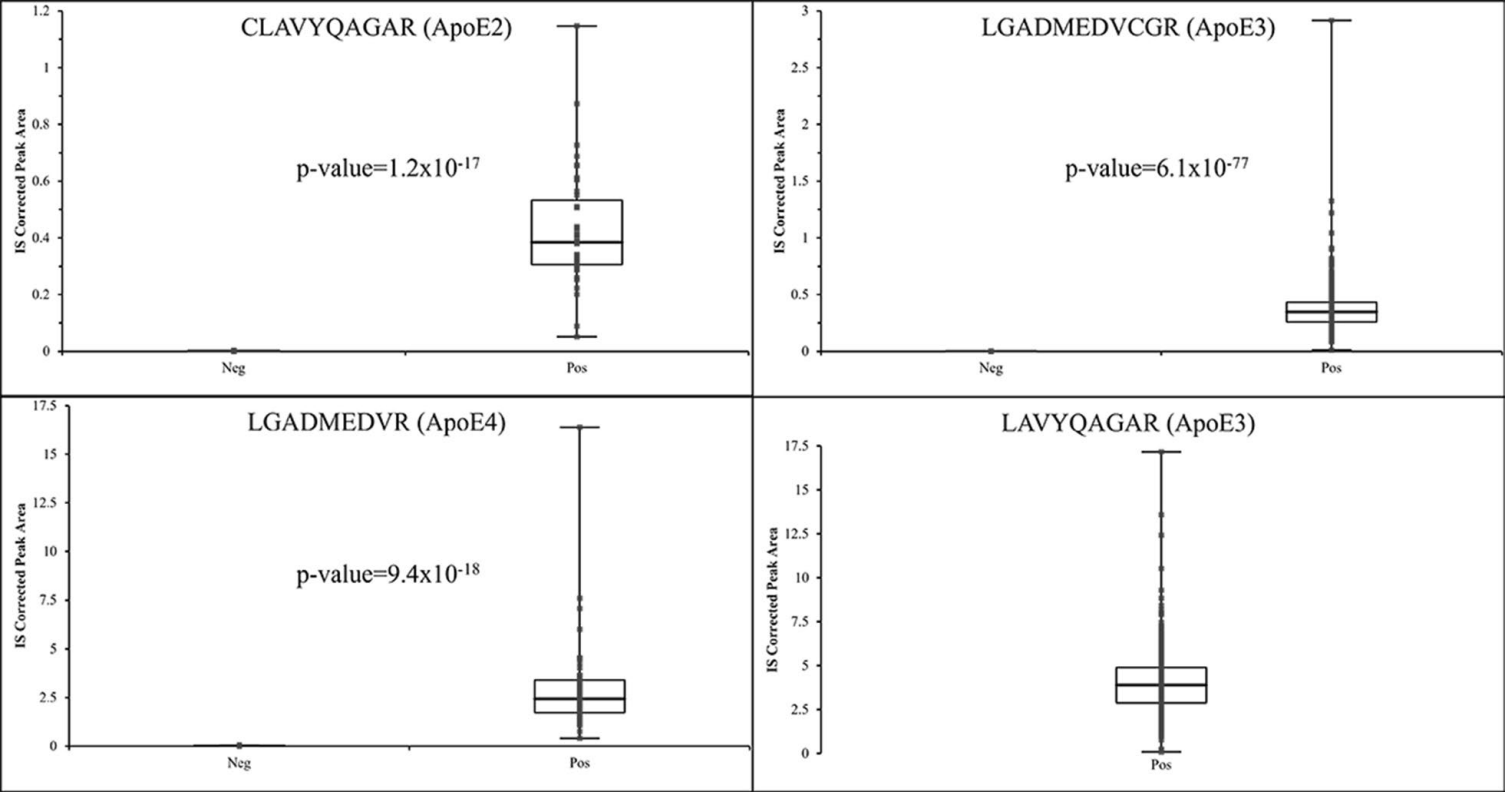
Box plots of the distribution of IS corrected peak areas derived from LS-MS/MS measurement of the ApoE peptides necessary for proteotyping. The p-value from a Welch’s t-test comparing the positive and negative peak area distributions is inset. No samples without LAVYQAGAR peptide were found in this investigation. As indicated by the p-value and box plots, there was a large discrepancy between signals assigned as positive and negative enabling differentiation. Box plots represent medians and quartiles with whiskers extending across the result distribution

A diagram summarizing workflows for the two different mass spectrometry-based approaches used in this investigation is shown in Fig. 1 (created in BioRender). Initial screening for a suitable sample set for intact protein analysis was done by performing trypsin digestion on 276 residual serum samples and the resulting peptides were measured using a Thermo TLX-4 coupled to a Sciex 7500. From this, a subset of 41 samples was selected to undergo intact protein analysis and genomic sequencing and the results are shown in Table 2. Example chromatograms for three patients with ApoE proteotypes of E3/E4 (patient 1), E2/E3 (patient 11), and E2/E4 (patient 16) are shown in Fig. 2. Box plots showing the resulting distribution of IS corrected peak areas for the 4 target peptides are shown in Fig. 3. Based on these plots, thresholds for determining positive and negative were set between these apparent distributions to assign positive and negative status for a given peptide and ultimately determine proteotype. When performing peptide analysis, differences in IS corrected intensity were very large between samples assigned as positive and negative. The closest difference between a positive and negative assignment was a factor of 8 in IS corrected area for the total set, and a factor of 25 for the smaller subset of 41 samples. The wide discrepancy is further evidenced by the very small p-values shown in Fig. 3. This large discrepancy between the positive and negative samples allowed for easy differentiation between the two distributions and ultimate assignment of the ApoE proteotype. All the samples exhibited a signal with a S/N ratio greater than 50 for peptide LAVYQAGAR; therefore, all samples were considered positive for this peptide. This peptide would only be absent in the case of ApoE2 homozygotes, which is the rarest possible genotype [2, 3]. Thus, our conclusion that our screening sample set possessed no ApoE2 homozygotes was plausible.

The selected subset of 41 samples then underwent IP followed by high-resolution LC–MS analysis on an Exploris 480. Signals from the intact protein analyses were processed using traditional integration of XICs and spectral deconvolution in BioPharma Finder. Example chromatograms from the aforementioned E3/E4, E2/E3, and E2/E4 patients are shown in Additional file 1: Fig. S2. These chromatograms are of relatively low S/N. The box plots of peak areas and coinciding p-values shown in Additional file 1: Fig. S3 also indicate a relatively small separation between the positive and negative samples, with only approximately a factor of 2 separating the positives and negatives for the ApoE3 and E4 proteins. These factors made accurately assigning the correct proteotype by this method more difficult.

**Fig. 4:**
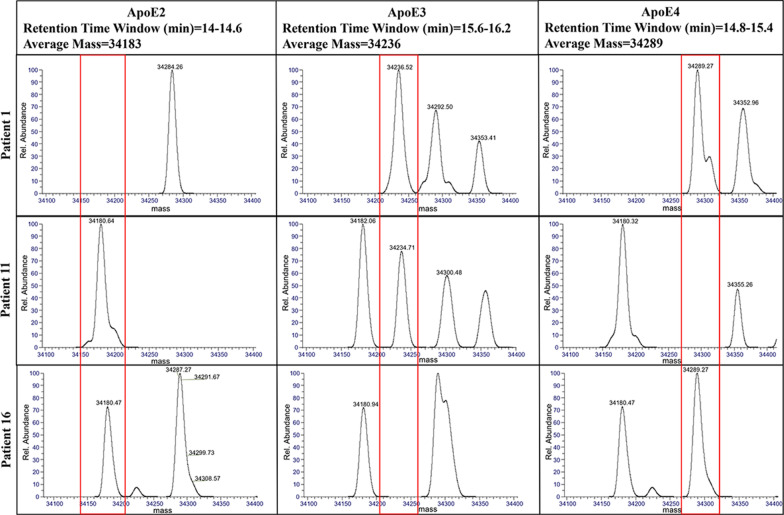
Deconvoluted spectra from three patients. The proteins were chromatographically separated, so a retention time window corresponding to the respective proteins was averaged to produce the deconvoluted spectrum. The red boxes correspond to the theoretical average mass of the proteins. As indicated in the spectra, patient 1 expressed the ApoE3 and ApoE4 proteins, patient 11 expressed the ApoE2 and ApoE3 proteins, and patient 16 expressed the ApoE2 and ApoE4 proteins

The benefits of using spectral deconvolution for detection of ApoE proteins were also explored. Deconvolution was done by averaging spectra in the chromatographic window corresponding to the three target proteins, which are chromatographically separated. Representative spectra from the aforementioned E3/E4, E2/E3, and E2/E4 patients are shown in Fig. 4. The deconvoluted spectra at the established retention times are compared to the theoretical average mass to determine the ApoE proteotype. Box plots of the deconvoluted spectral signal intensities are shown in Additional file 1: Fig. S4. Similar to the peptide approach described above, intensity thresholds were set between the apparent positive and negative distributions for the respective proteins. The minimum separation between positive and negative assignments was observed for E4, which differed by a factor of 3.7 and resulted in the relatively large p-value of 1.88 × 10^–2^.

The subset of 41 samples that underwent both trypsin digestion and subsequent intact protein characterization were also analyzed by ApoE genomic sequencing in order to compare assessment by genotyping and proteotyping methods (Table 2). The accuracy of these mass spectrometry-based techniques compared to the genomic sequencing are shown in Table 3. The chromatographic integration of LC–MS signals was the least accurate methodology with three discrepant results compared to genomic sequencing likely due to the factors discussed previously. Spectral deconvolution resolved one of the discrepancies of the intact method in comparison to sequencing results. Peptide analysis resulted in only a single discrepancy when compared to the genomic sequencing results. The results for this discrepant sample matched for all of the mass spectrometry-based techniques, but were contradictory to the sequencing results. The sample was reanalyzed by the peptide technique and yielded the same result, but there was insufficient sample for repeat genomic sequencing. Therefore, the reason for the discrepancy is unknown. If deconvolution is utilized to process the intact protein spectra, both the peptide and the intact method mischaracterized the same E3/E4 genotype sample as an E3/E3 and the intact method mischaracterized an additional E3/E4 genotype sample as an E3/E3 proteotype sample.

**Table 3:** Percent accuracy of the mass spectrometry-based techniques relative to genomic sequencing

Genotype	Number of samples (based on genomic sequencing)	% Accuracy intact protein deconvolution	% Accuracy intact protein chromatogram	% Accuracy peptide
E2/E2	0	100%	100%	100%
E2/E3	6	100%	100%	100%
E3/E3	18	100%	100%	100%
E3/E4	9	78%	78%	89%
E2/E4	3	100%	66%	100%
E4/E4	2	100%	100%	100%

Overall, this work demonstrates the viability of intact protein workflows for LC–MS proteotyping of ApoE. Additionally, the comparison of chromatographic and deconvolution-based approaches is relevant to clinical laboratories developing tests targeting intact proteins. Most clinical mass spectrometry tests are predicated on the integration and quantitation of chromatograms; whereas the field of intact proteomics widely relies on deconvolution for qualitative analyses. As quantitative clinical analyses of larger proteins become more prevalent, the benefits, precision, and accuracy of deconvolution must be carefully vetted and clearly demonstrated. Our data indicates that deconvolution improves the S/N and coincidingly the ability to characterize the proteins, which aligns with research applications. Assessing the long-term quantitative performance was beyond the scope of this work.

Intact protein analysis clearly has several limitations. Most importantly, in the small sample set analyzed in this investigation, both data processing techniques resulted in more discrepancies compared to genomic sequencing than did the peptide analysis method. This can be attributed to the lower sensitivity to the presence of ApoE of the intact protein technique compared to the peptide detection method. Intact protein analysis yielded lower S/N, as evidenced by the reduced difference between the positive and negative samples, generally lower p-values, and chromatograms. To overcome the S/N challenges with intact protein analysis, a large quantity of antibody was used to increase the signal. We initially tried 5 µg as described by Hu and coworkers [27], but found our methodology benefited from the approximately 2 × gain in S/N when using 2 times more antibody (data not shown). However, the cost of this strategy would likely be prohibitive in a routine testing environment. A state-of-the-art high-resolution mass spectrometer was also used for the intact protein analysis. Although high-resolution mass spectrometers are becoming more common in clinical labs, they are not nearly as well established as the triple quadripole mass spectrometer used for peptide analysis, and therefore, not as preferable.

Potential differences in the run-time and approximate cost should be considered when selecting a viable ApoE proteotyping method. Intact protein analysis requires IP from 500 μL of sample and two hours of sample preparation. This process can be automated, but was quite costly as 10 μg of antibody was required to yield a sufficient signal-to-noise (S/N) ratio. Peptide analysis requires only 25 μL of sample and is relatively inexpensive. The sample preparation is slightly more time consuming and laborious; however, the higher S/N of the resulting peptide signals allowed for a 2 × decrease in chromatographic separation time and several commercial platforms are available for automation of these types of procedures. A summary comparison of the materials cost, sample preparation time, and analysis time can be seen in Table 4. This clearly demonstrates the financial advantages of the peptide analysis method, as the list price of the materials for protein measurements cost $55.00 and sequencing was $36.02, whereas the peptide measurement materials were only $0.08. Based on the publication by Hu et al., MALDI-TOF–MS yields higher S/N, but would still require expensive antibody-based purification and the resulting spectra would be difficult to interpret in the routine clinical laboratory due to the lack of chromatographic and mass resolution.

**Table 4:** Comparison of the materials cost based on list prices, sample preparation time, and analysis time estimates when performing peptide, protein, and geomic analyses

	Peptide analysis	Intact protein analysis	Genomic sequencing
Materials Cost per Sample ($)	0.08	55.00	36.02
Sample Preparation Time (H)	2.5	2	2
Instrument Analysis Time (min)	3.125	7.5	155

Another significant challenge when performing analysis of intact proteins such as ApoE is finding an appropriate internal standard to compensate for variation in instrument performance and sample preparation. Isotopically labeled peptides are easily synthesized for relatively low cost. However, isotopically labeled proteins are often not commercially available or are prohibitive from a cost perspective. This investigation provides a quintessential example of these challenges. The ApoE peptide analysis utilized isotopically labeled peptides, which makes the analysis more reproducible overall. These implications are magnified when setting thresholds for positive and negative as done in this work. The use of isotopically labeled internal standards would make the use of a positive/negative intensity threshold more robust over the short and long term. In contrast, no feasible internal standard material was found for the intact protein analysis. Even if an isotopically labeled protein existed, if one added it prior to the antibody capture this would reduce the capacity for the target protein and lower the LC–MS signal from the protein. Therefore, a myriad of factors (primarily the lack of availability) made the use of an internal standard impractical for the intact protein analysis measurement. This likely contributes to the comparatively lower performance of the protein analysis observed herein, and would likely negatively impact the ability to apply positive/negative thresholds over the long term.

The peptide analysis methodology presented within this study has several advantages over methods described in previous publications. The sample preparation is completed in approximately 2.5 h and is highly amenable to automation. The multiplex analytical flow LC and triple quadrupole mass spectrometer used are widely available in routine clinical laboratories today, well suited for online sample purification, and are capable of producing results approximately every 3 min. Although quantitation was not a goal of our work, with the addition of an external calibration curve this methodology would be well-suited to produce accurate and precise quantitative results.

This investigation has several limitations. Due to the relatively high cost of genomic and intact protein analyses, only a subset of the initial 276 samples could be analyzed by these techniques. Based on the initial screening, a sample set was selected that disproportionately represented the lower frequency genotypes, but it would be ideal to have a larger sample set with a wider variety of genotypes, including an ApoE2 homozygous sample, one of which was not identified in the initial screening. It would also be advantageous to obtain another sample from the subject that produced the discrepancy between the mass spectrometry and genomic methodologies. Performing a longer-term study to assess the performance of these methodologies and the applicability of the thresholds established herein over time would also be beneficial, but such an effort is beyond the scope of this work. Use of an external calibrator to yield quantitative results and to establish thresholds as opposed to signal intensity metrics would also be a possible improvement for future work.

## Conclusions

Our results demonstrate that measurement of intact ApoE is a viable means of proteotyping. We used immuno-purification followed by high-resolution LC–MS analysis and spectral deconvolution to yield results that were in over 90% agreement with established genomic sequencing techniques. However, when compared to the high throughput, inexpensive peptide analysis method developed on the triple quadrupole mass spectrometer, the intact protein method is less accurate, more costly, more laborious, less robust, and requires more advanced instrumentation. Therefore, until LC–MS technology advances clinical laboratories are much better served to perform these types of analyses on digested peptides.

## Supplementary Information

**Additional file 1: Table S1.** Sciex 7500 source conditions.** Table S2.** Sciex 7500 parameters.** Table S3.** Exploris 480 parameters.** Fig. S1.** Box plots of the distribution of IS corrected peak areas derived from LC-MS/MS measurement of the ApoE peptides necessary for proteotyping for only the subset of samples selected for subsequent analyses.** Fig. S2.** Representative chromatograms of the intact protein XICs.** Fig. S3.** Box plots of the distribution of peaks areas from the integration of chromatographic peaks when performing LC-MS measurement of intact ApoE.** Fig. S4.** Box plots of the distribution of deconvoluted spectral signal intensities when performing LC-MS measurement of intact ApoE.

## Data Availability

The mass spectrometry data have been deposited in the PeptideAtlas SRM Experiment Library with the data set identifier PASS01770.

## Abbreviations

ApoE: Apolipoprotein E
VLDL: Very low density lipoprotein
HDL: High density lipoproteins
AD: Alzhiemer’s disease
LC–MS: Liquid chromatography mass spectrometry
LC–MS/MS: Liquid chromatography-tandem mass spectrometry;
PBS: Phosphate Buffered Saline
ACN: Acetonitrile
IPA: Isopropanol
BSA: Bovine serum albumin
IS: Internal standards
FMOC: Fluorenylmethoxycarbonyl
TFA: Trifluoroacetic acid
XIC: Extracted ion chromatogram
S/N: Signal-to-noise
