# Supplementary material for: Comparison of intact protein and digested peptide techniques for high throughput proteotyping of ApoE
Source: Clin Proteomics. 2022 Nov 15;19:42. doi: 10.1186/s12014-022-09379-5 (PMC9664673; doi:10.1186/s12014-022-09379-5)
Supplement: Supplementary file 1 — Additional file 1: Table S1. Sciex 7500 source conditions. Table S2. Sciex 7500 parameters. Table S3. Exploris 480 parameters. Fig. S1. Box plots of the distribution of IS corrected peak areas derived from LC-MS/MS measurement of the ApoE peptides necessary for proteotyping for only the subset of samples selected for subsequent analyses. Fig. S2. Representative chromatograms of the intact protein XICs. Fig. S3. Box plots of the distribution of peaks areas from the integration of chromatographic peaks when performing LC-MS measurement of intact ApoE. Fig. S4. Box plots of the distribution of deconvoluted spectral signal intensities when performing LC-MS measurement of intact ApoE. [file 12014_2022_9379_MOESM1_ESM.docx]

**Supplementary Information for**

**Comparison of Intact Protein and Digested Peptide Techniques for High Throughput, Inexpensive Proteotyping of ApoE**

Anthony Maus^1*^, Dan Figdore^1^, Dragana Milosevic^1^, Alicia Algeciras-Schimnich^1^, Joshua Bornhorst^1^

^1^Department of Laboratory Medicine and Pathology, Division of Clinical Biochemistry and Immunology, Mayo Clinic, Rochester, MN 55905, USA

* **Corresponding Author:** Anthony Maus, 200 First Street SW, Rochester, MN 55905, Maus.anthony@mayo.edu, 507-538-0429

| Polarity | Pos |
| --- | --- |
| Spray Voltage | 3000 |
| GS1 (PSI) | 75 |
| GS2 (PSI) | 75 |
| Curtain Gas (PSI) | 40 |
| Source Temp (°C) | 450 |

**Table S1.** Sciex 7500 source conditions

| Name | Q1 mass | Q3 mass | Dwell Time (ms) | EP (V) | CE (V) | CXP (V) | Q0D (V) |
| --- | --- | --- | --- | --- | --- | --- | --- |
| CLA-1 | 554.8 | 835.4 | 25 | 10 | 30 | 15 | 50 |
| CLA-2 | 554.8 | 764.4 | 25 | 10 | 30 | 15 | 50 |
| CLAIS-1 | 559.8 | 845.5 | 25 | 10 | 30 | 15 | 50 |
| CLAIS-2 | 559.8 | 774.4 | 25 | 10 | 30 | 15 | 50 |
| CGR-1 | 611.8 | 1052.5 | 25 | 10 | 33 | 15 | 50 |
| CGR-2 | 611.8 | 735.4 | 25 | 10 | 33 | 15 | 50 |
| CGRIS-1 | 616.8 | 1062.5 | 25 | 10 | 33 | 15 | 50 |
| CGRIS-2 | 616.8 | 745.4 | 25 | 10 | 33 | 15 | 50 |
| LAV-1 | 474.8 | 764.5 | 25 | 10 | 27 | 15 | 50 |
| LAV-2 | 474.8 | 665.4 | 25 | 10 | 27 | 15 | 50 |
| LAVIS-1 | 479.8 | 774.5 | 25 | 10 | 27 | 15 | 50 |
| LAVIS-2 | 479.8 | 675.4 | 25 | 10 | 27 | 15 | 50 |
| DVR-1 | 503.3 | 835.4 | 25 | 10 | 28 | 15 | 50 |
| DVR-2 | 503.3 | 764.4 | 25 | 10 | 28 | 15 | 50 |
| DVRIS-1 | 508.3 | 845.4 | 25 | 10 | 28 | 15 | 50 |
| DVRIS-2 | 508.3 | 774.4 | 25 | 10 | 28 | 15 | 50 |

**Table S2.** Sciex 7500 parameters

| Polarity | Pos |
| --- | --- |
| Spray Voltage (V) | 3500 |
| Sheath Gas (Au) | 50 |
| Aux Gas (Au) | 10 |
| Sweep Gas (Au) | 1 |
| Ion Transfer Tube Temp (°C) | 325 |
| Vaporizer Temp (°C) | 375 |
| Orbitrap Resolution | 240000 |
| Scan Range (m/z) | 1000-3000 |
| Time (ms) | 500 |
| Microscans | 1 |
| AGC Target | Standard |
| RF Lens% | 60 |

**Table S3.** Exploris 480 parameters


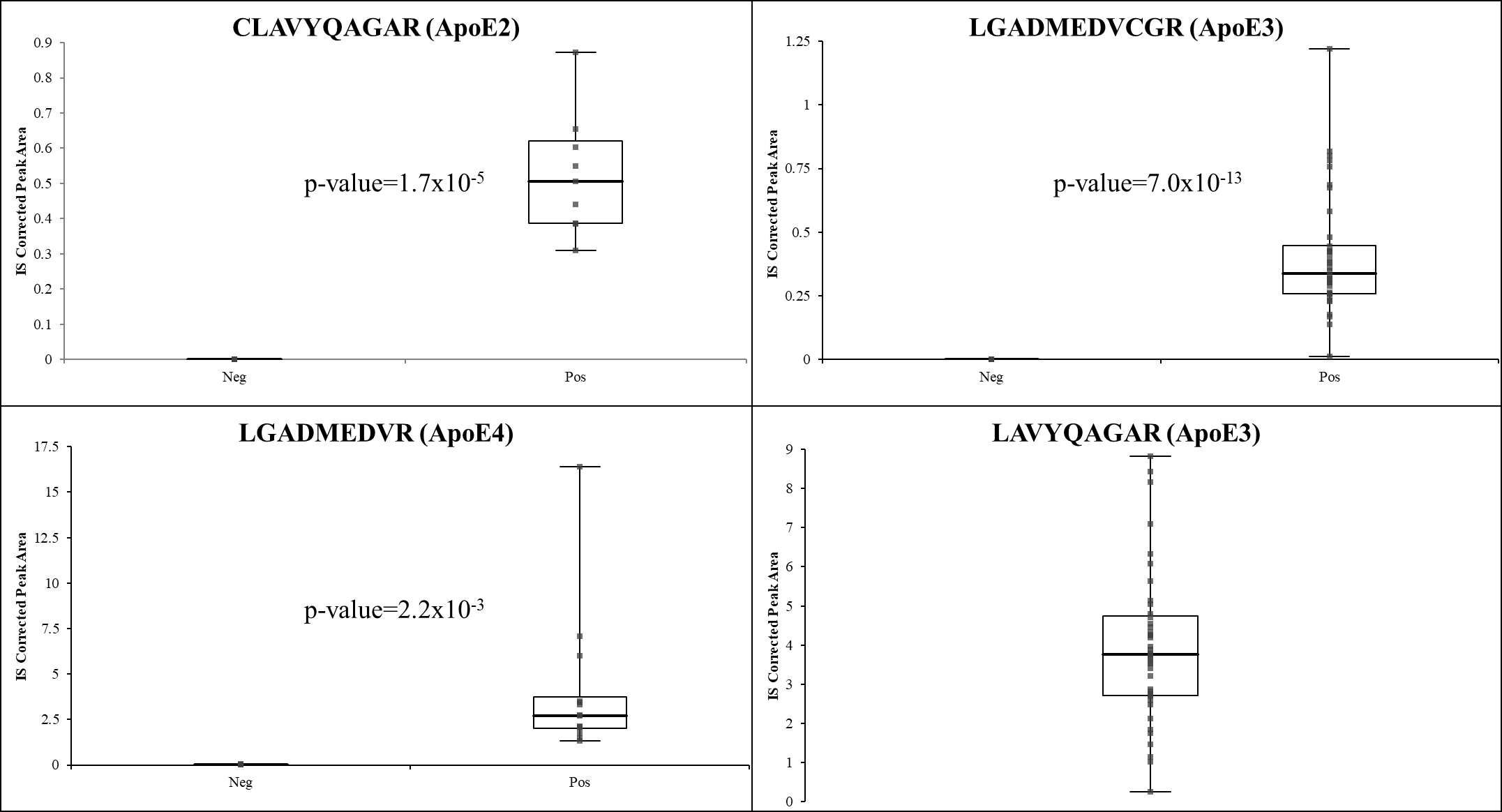


**Fig. S1.** Box plots of the distribution of IS corrected peak areas derived from LC-MS/MS measurement of the ApoE peptides necessary for proteotyping for only the subset of samples selected for subsequent analyses. The p-value from a Welch’s t-test comparing the positive and negative peak area distributions is inset. As indicated by the p-value and box plots, there was a large discrepancy between signals assigned as positive and negative enabling differentiation.


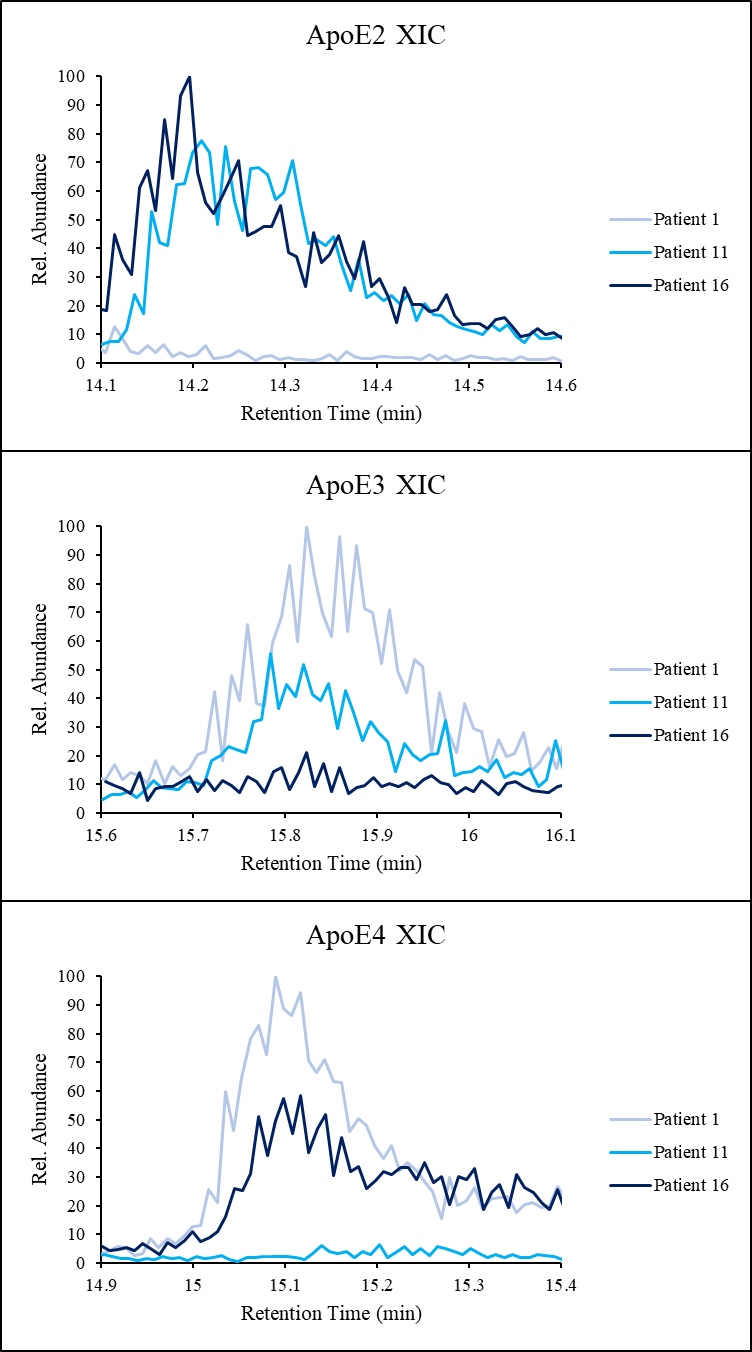


**Fig. S2.** Representative chromatograms of the intact protein XICs. Patient 1 expressed the ApoE3 and ApoE4 proteins, Patient 11 expressed the ApoE2 and ApoE3 proteins, and patient 16 expressed the ApoE2 and ApoE4 proteins.


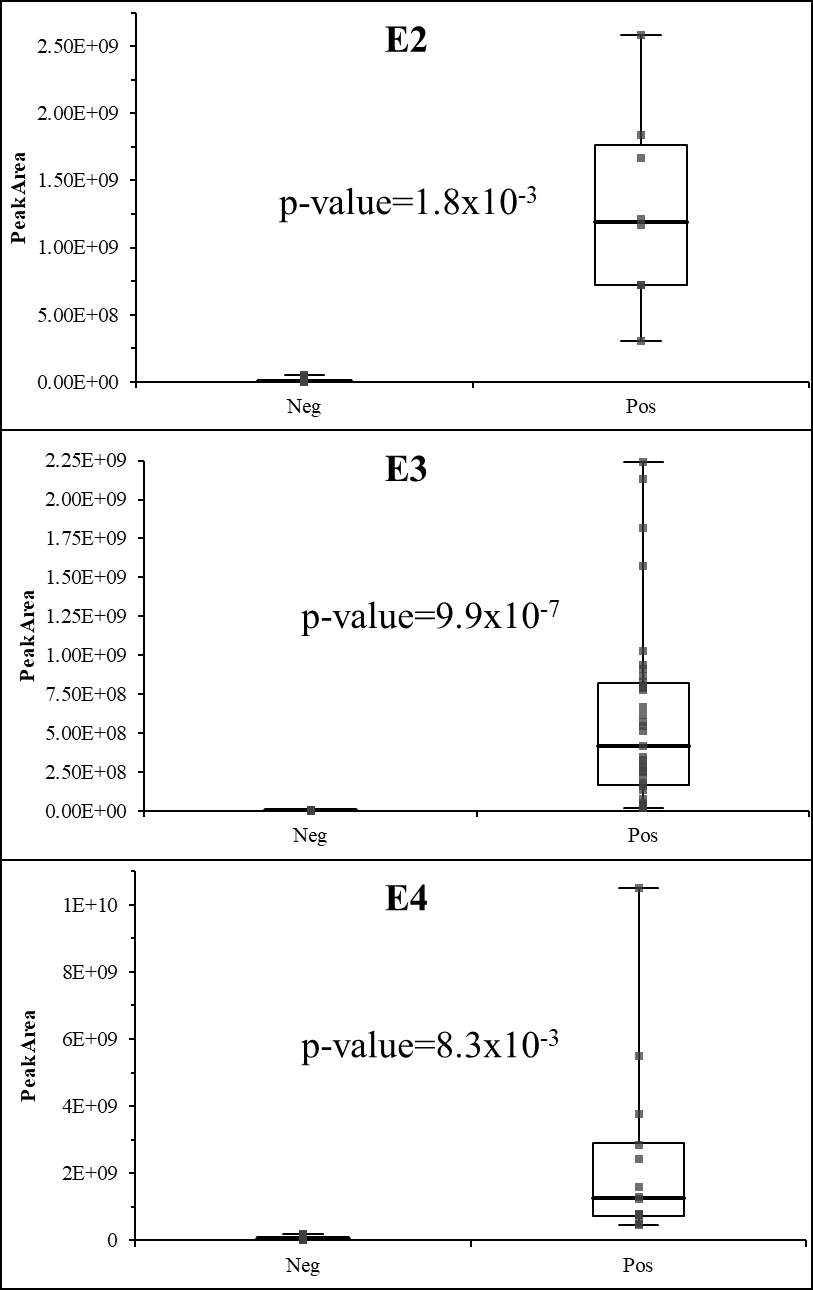


**Fig. S3.** Box plots of the distribution of peaks areas from the integration of chromatographic peaks when performing LC-MS measurement of intact ApoE. The p-value from a Welch’s t-test comparing the positive and negative peak area distributions is inset.


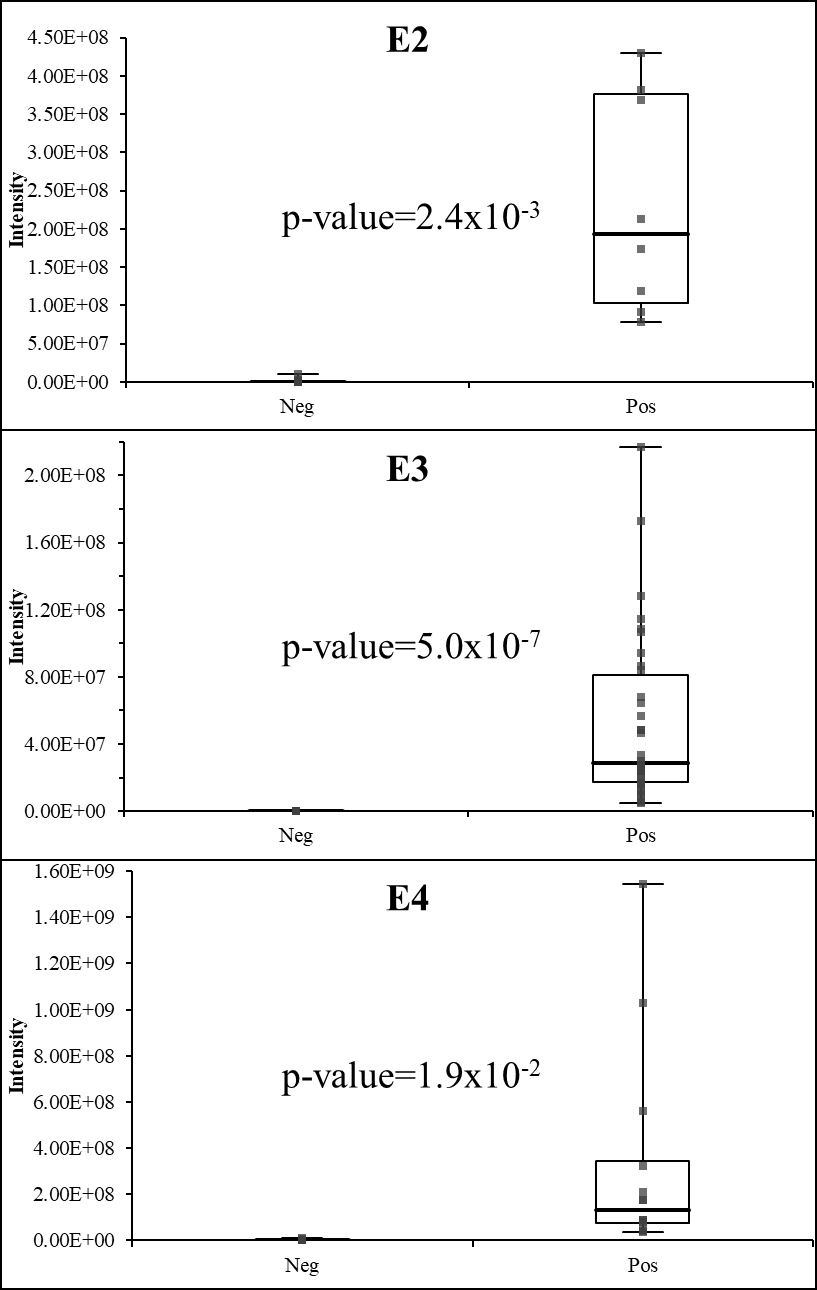


**Fig. S4.** Box plots of the distribution of deconvoluted spectral signal intensities when performing LC-MS measurement of intact ApoE. The p-value from a Welch’s t-test comparing the positive and negative peak area distributions is inset.
